# Comparative metavirome analysis in polytransfused patients

**DOI:** 10.1590/1414-431X2021e11610

**Published:** 2021-10-18

**Authors:** I.N. Valença, F.A. Rós, V.S. Zucherato, A.C. Silva-Pinto, D.T. Covas, S. Kashima, S.N. Slavov

**Affiliations:** 1 Programa de Pós-Graduação em Oncologia Clínica, Células Tronco e Terapia Celular, Faculdade de Medicina de Ribeirão Preto, Universidade de São Paulo, Ribeirão Preto, SP, Brasil; 2 Hemocentro de Ribeirão Preto, Faculdade de Medicina de Ribeirão Preto, Universidade de São Paulo, Ribeirão Preto, SP, Brasil; 3 Instituto Butantan, São Paulo, SP, Brasil

**Keywords:** Virome, Metagenomic analysis, Multiple transfusions, Beta-thalassemia major, Sickle cell disease

## Abstract

Due to the high transfusion volume, polytransfused patients with sickle cell disease (SCD) and beta-thalassemia are constantly exposed to parenterally transmitted infections. Currently, we have little information about the virome of such patients and how the virological composition might be influenced by the hemotherapy procedures that these patients receive. The objective of this study was to compare the viral diversity between these two groups with respect to the viral abundance and how it might be affected by the specific conditions of these groups. We sequenced by next-generation sequencing (NGS) and compared the virome of 30 patients with beta-thalassemia major, 45 with SCD, and 16 blood donors from the Blood Center of Ribeirão Preto, Brazil. Predominantly, commensal viruses including Torque teno virus (TTV) genotypes and human pegiviris-1 (HPgV-1) were identified in each group. Strikingly, while HPgV-1 reads were dominant in the SCD group, thalassemic patients showed high TTV abundance, expressed both in viral reads and genotypes. We speculated that the commensal virome of polytransfused patients might be influenced by the transfusion frequency and disease characteristics and that commensal viruses might be used as important genetic biomarkers for these hematological disturbances. Nevertheless, more specific studies are necessary to confirm a relationship between blood virome and transfusion treatment.

## Introduction

Blood transfusions are an efficient and increasingly safe treatment option for patients with blood cell disorders. In patients with hemoglobinopathies (thalassemia syndromes, structural hemoglobin variants) blood transfusions supply healthy red blood cells (RBC) that temporarily correct anemia and relieve clinical complications like inefficient erythropoiesis, RBC pathologic conformation, inflammation, and hemolysis (1). Despite the diagnostic advances regarding routinely tested blood-borne infections, the high number of transfusions in patients with hemoglobinopathies poses an increased risk for acquiring parenteral infections, especially for those who are not routinely tested. Moreover, patients with beta-thalassemia have higher hepatitis C virus (HCV) seroprevalence rates (2), which additionally suggests that the high transfusion burden might be related to higher rates of parenteral infections in this high-risk group. It is now accepted that the introduction of routine and highly sensitive tests for detection of blood-borne infections has drastically reduced their transmission rates among polytransfused patients as observed in a recently published survey, where the HCV-seroprevalence was 2.5% in older patients with sickle cell disease (SCD) (3), which indicates that HCV was acquired before the routine testing for this infection (4). Nevertheless, there is always the risk of residual transmission due to factors including extremely high viral loads, mutations in the target sequence, and window period testing in which infection markers are undetectable (5). Apart from this, virtually any virus that establishes viremia is transmissible by blood transfusion including even exotic viral agents like those normally transmitted by arthropods such as Japanese encephalitis (6), Dengue (7), and the Powassan virus (8), of which transfusion transmission has also been documented and may equally affect polytransfused patients.

The objective of this study was to compare the virome composition of patients with beta-thalassemia major and SCD patients who receive multiple transfusions. Our analysis was driven from the observation that the frequency of blood transfusions may influence the virome abundance and that specific viral agents that compose the virome of these patients may be used as hypothetical markers for characterizing underlying diseases.

## Material and Methods

### Patient groups, demographic and clinical data

In this study, we compared the viral profile in two groups of patients who received multiple transfusions: 30 patients with beta-thalassemia and 45 with SCD. The SCD group included 23 male (51.1%) and 22 female (48.9%) patients with a mean age of 26.2 years (range 8-57 years). The beta-thalassemia group comprised 20 male (66.7%) and 10 female (33.3%) patients with a mean age of 26.6 years (range 6-47 years). In addition, we also reviewed the transfusion history of the patients (number of transfused blood units, volume of transfused blood, and body weight of the patients during the year prior to sample collection). In beta-thalassemia patients, the following prevalence of common transfusion-transmitted infection markers was observed: 23.3% (n=7/30) of the patients were anti-HCV IgG positive, 16.7% (n=5/30) were positive for anti-HBsAg, and 3.3% (n=1/30) were positive for anti-HBc total antibodies. In the SCD patient group, the seroprevalence of routine blood-transmitted infections revealed four cases positive for HBV serological markers (8.9%; one case of anti-HBc IgG, one case of HBsAg, and two cases of anti-HBsAg), one case of HCV (2.2%), and one case of Chagas disease (2.2%). Finally, we used a control group consisting of 16 blood donors, whose samples were also analyzed by next-generation sequencing (NGS). The demographic characteristics of the healthy blood donors were the following: mean age of 40.5 years (range 26-58 years) including 8 women (50%) and 8 men (50%). All blood donors who participated in this study were eligible for blood donation as they were approved at all interviews and had not been transfused in the previous year. This study was approved by the Institutional Ethics Committee of the University Hospital at the Faculty of Medicine of Ribeirão Preto, University of São Paulo, Brazil (process number, HCRP-12196/2018).

### Sample preparation and Illumina NextSeq 500 sequencing

Six hundred μL of patient plasma were pre-treated with Turbo DNAse (Turbo DNA-free kit, ThermoFisher Scientific, Brazil) aiming host/bacterial DNA removal. After DNAse inactivation, 5 to 6 individual samples were mixed in a single pool. The total pool quantity was extracted using the High Pure Viral Nucleic Acid Large Volume kit (Roche, Brazil) following the manufacturer's instructions. The extracted samples were submitted to reverse transcription using the Superscript III First-Strand Synthesis System (ThermoFisher Scientific). The amplification was performed using the QuantiTect Whole Transcriptome kit (QIAGEN, Brazil). Genomic libraries were prepared using the Nextera DNA Flex Library Preparation and Nextera DNA CD Indexes kits (Illumina, USA). The sequencing was performed in Illumina NextSeq 500 sequencer using the NextSeq High Output Kit v 2.5, 300 cycles (Illumina).

### Bioinformatics analysis

Sequence reads were initially processed to examine their quality using the FastQC v.0.11.8 software (available online at: http://www.bioinformatics.babraham.ac.ujk/projects/fastqc). The low-quality reads, sequences with ambiguous bases (*N*), and duplicated reads were removed using the Trimmomatic v.0.36 software (usadellab.org). To remove host-related sequences, the DeconSeq (v.0.4.3) software (https://sourceforge.net/projects/deconseq/files/) was applied. The remaining sequences were taxonomically analyzed using Kraken2 (https://ccb.jhu.edu/software/kraken2/). The unclassified reads were initially analyzed through Blastx and Blastn (blast.ncbi.nlm.nih.gov) against a database composed only of viral sequences. Potential viruses of interest were *de novo* assembled using SPAdes v.3.13.0 software (cab.spbu.ru) and analyzed manually. All generated contigs were analyzed by Blastn using a nucleotide database downloaded by the NCBI and an E-value cutoff of 10^-3^. The protein similarity was evaluated using Blastx.

### Molecular detection of HPgV-1 RNA

HPgV-1 RNA detection was performed by nested-PCR using primers described in the literature (9), which were able to detect all HPgV-1 genotypes targeting a highly conserved portion of the 5′-UTR region of the HPgV-1 genome. The following protocol was used: denaturation at 95°C for 5 min, followed by 35 cycles of 95°C for 30 s, 55°C for 30 s, and 72°C for 1 min. The PCR reaction was finalized using a final extension at 72°C for 10 min. The second reaction was performed using the same protocol with the difference that 40 cycles were applied. The obtained amplicons were visualized in 2% agarose gel.

## Results

### Transfusion profile of beta-thalassemic and SCD patients

**Figure 1:**
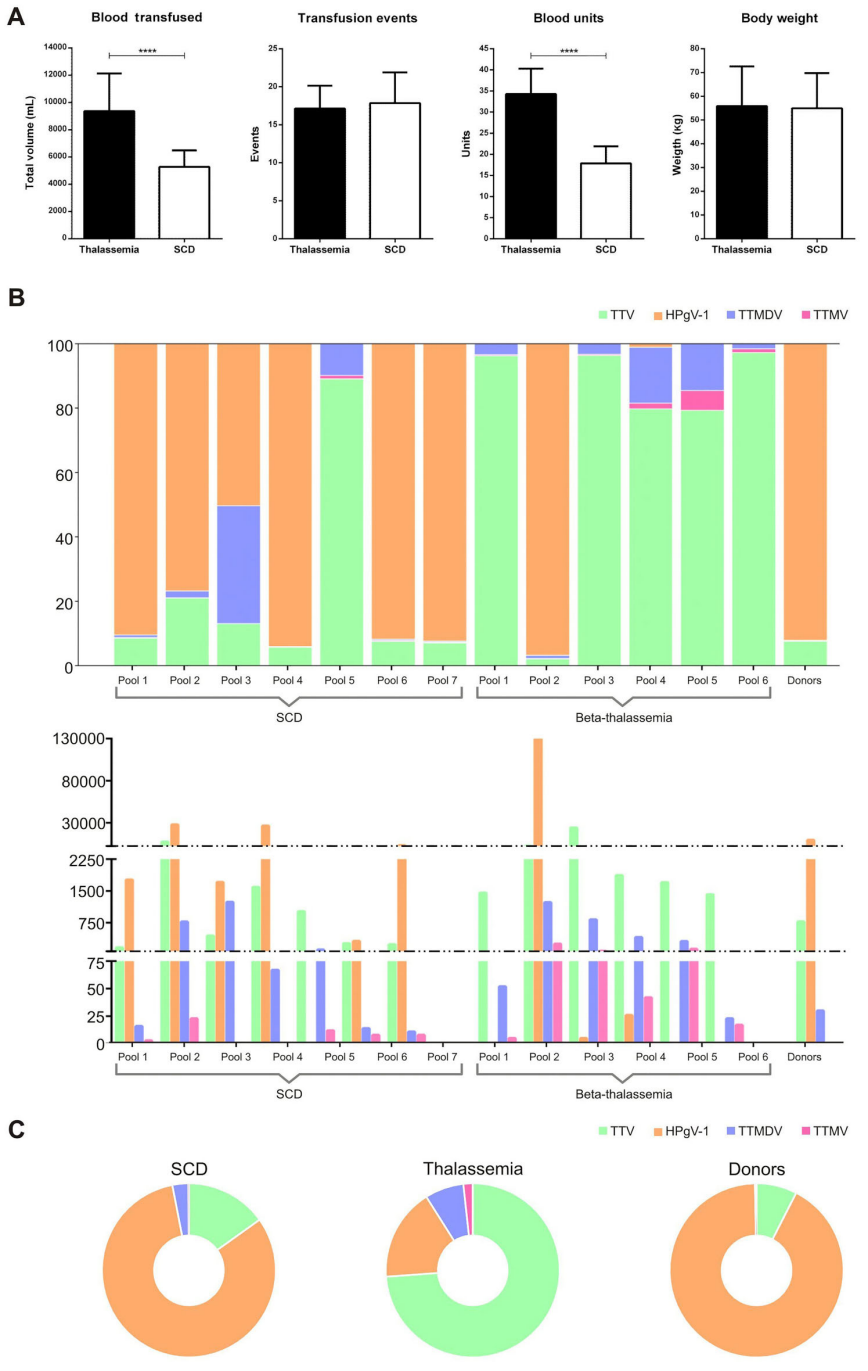
**A**, Clinical characteristics of the patients with sickle cell disease (SCD) and beta-thalassemia major. Data are reported as means±SD. ****P<0.001 (*t*-test). **B**, Virome composition (%, upper chart; number, lower chart) of the patients with SCD and beta-thalassemia major by each pool (SCD 1 to 7 and beta-thalassemia 1 to 6). **C**, Mean percentage of the reads for TTV, TTMDV, TTMV, and HPgV-1 in the pools of the two patient groups and negative control (blood donors).

We calculated the average volume of transfused blood for a one-year period (October, 2017 to October, 2018) that was received by the thalassemic and SCD patients. Regarding the number of transfusion events (TE), we calculated an average of 17.84±3.99 TE among patients with beta-thalassemia and 17.13±2.95 in SCD patients. The average volume of transfused packed red blood cells (blood units) in patients with beta-thalassemia major was 9358.46±2724.4 mL and these patients showed an average body weight of 55.8±16.42 kg. For the group of SCD patients we observed a lower average volume of 5277.84±1194.66 mL and a similar average body weight of 54.9±14.69 kg (Figure 1A). The thalassemic patients received an average of 34 blood units while the SCD group had an average of approximately 18 blood units. These blood units were estimated by the average content of a single blood unit (approximately 250∼300 mL) and the average volume of transfusion events for each group.

### Virome comparison between the groups

In pools of patients with beta-thalassemia major we identified predominantly commensal viruses. Two major viral specimens were identified: HPgV-1 (*Flaviviridae* family) and different representatives of the TTV family. TTV 11 (3477 reads) and TTV 19 (3111 reads) were the most abundant anellovirus species in the SCD group while TTV 12 (1486 reads) and also TTV 11 (1145 reads) were the most representative for the thalassemic group. Among the 7 pools from the SCD group, 6 showed a high representation of HPgV-1 reads while among the 6 pools from thalassemic patients, 5 showed a major representation of the *Anelloviridae* family (Table 1). Strikingly, while HPgV-1 was practically absent in the thalassemic group, the only positive pool for that virus (pool 2) showed the highest number of HPgV-1 reads considering both SCD and beta-thalassemia patients. The control group showed a viral distribution similar to the SCD group, where HPgV-1 (9530 reads) significantly outnumbered the presence of TTVs (910 reads) (Figure 1B and C). In the pools of patients with beta-thalassemia, the metagenomics analysis also identified the presence of pathogenic viruses like HCV and HBV, which was expected due to the observed seroprevalence to both viral agents in this patient group.

**Table 1:** Anellovirus genomic diversity and read counts among patients with sickle cell disease, beta-thalassemia major, and blood donors.

Anellovirus diversity					
Sickle cell disease		Beta-thalassemia		Blood donors	
Type	Read count	Type	Read count	Type	Read count
TTV 12	1486	TTV 11	3477	TTV 24	198
TTV 11	1145	TTV 19	3111	TTV 13	68
TTV 10	920	TTV 6	2863	TTV 19	54
TTV 15	707	TTV 13	2768	TTV 15	43
TTV 19	601	TTV 10	1964	TTV 10	32
TTV 6	572	TTV 3	1498	TTV 3	27
TTV 8	380	TTV 15	1377	TTV 12	27
TTV 5	363	TTV 12	1115	TTV 16	26
TTV 24	344	TTV 7	995	TTV 5	25
TTV 3	315	TTV 16	952	TTV 1	23

### Prevalence of HPgV-1 among groups

Due to the presence of a representative number of viral reads belonging to HPgV-1, we tested individual samples composing each pool. From a total number of 30 plasma samples obtained from patients with beta-thalassemia, 2 were positive for HPgV-1 RNA (n=2/30, 6.7%). Interestingly, these samples belonged to the same pool (pool No. 2) that was positive for HPgV-1 RNA among all tested pools of the patients with beta-thalassemia. In SCD patients, we detected 8 plasma samples positive for HPgV-1 RNA, which was also depicted by the high number of HPgV-1 reads detected in almost all tested pools obtained from SCD patients (6 out of 7 tested pools). The prevalence of HPgV-1 RNA among patients with SCD was significantly higher compared to patients with beta-thalassemia (n=8/45, 17.8%) (P<0.5). We also tested the prevalence of HPgV-1 RNA among healthy blood donors. We detected two positive samples among the 16 tested (n=2/16, 12.5%).

## Discussion

In this study, we compared the virome of two groups of polytransfused patients, i.e., beta-thalassemia major and SCD, aiming to characterize viruses with impact on transfusion safety and also to identify some viruses that may serve as viral-specific molecular markers of both diseases. The choice of these groups in our study was based on the elevated transfusion frequency and the quantity of blood associated with the lifetime treatment of beta-hemoglobinopathies.

In both patient groups and in blood donors, a pronounced presence of commensal viruses and especially anelloviruses was found. Interestingly, the most abundant anellovirus species were shared between the patients with beta-thalassemia and SCD. Among the 10 most abundant TTV species, at least 5 were shared i.e., TTV 6, 10, 11, 12, 15, and 19. Nevertheless, although the anellovirus composition was similar, the quantitative read abundance was highly divergent between the studied groups. Patients with beta-thalassemia demonstrated a three-fold higher presence of TTV reads compared to SCD patients and blood donors. This may indicate that the quantitative rather than qualitative TTV abundance might be an important marker that characterizes polytransfused patients. In this respect, we believe that the number of transfused blood units is a leading cause for the higher anellovirus genotype abundance in beta-thalassemia patients. This was also supported by the transfusion profile analysis, which demonstrated that patients with beta-thalassemia major received a higher number of transfusion events, which may increase both TTV burden and genetic variety. The received transfusions might be responsible for many TTV infections or reinfections by different genotypes. This, added to the persistent TTV infections, may lead to the elevated number of TTV reads in patients with beta-thalassemia compared to SCD patients. On the contrary, SCD patients who did not receive transfusions showed a lower number of TTV reads, despite the higher number of patients included in this group. This was also observed in healthy blood donors, where the number of TTV reads was the lowest. Therefore, similar to transplant recipients in whom the anellovirus load has been suggested as a marker of immune functioning (10), we believe that TTV read abundance can be used as a surrogate marker for transfusion frequency. Although the TTV composition was similar between the patients, the most common TTVs in the groups were different. While TTV-12 was the most abundant type in SCD, TTV-11 was the most common in patients with beta-thalassemia. In blood donors, the most abundant TTV type was 24. Nevertheless, the obtained TTV composition in patients and blood donors seems to be similar to other studies that examined plasma virome not only of blood donors (11) but also of patients undergoing solid organ transplantation (12) and in kidney donors and recipients (13). Based on the above cited studies and the obtained results from our study, we believe that some TTV types might be more broadly adapted to the human blood environment despite the clinical condition of the host.

We also identified the commensal virus HPgV-1, which was represented differently among the three studied groups. While in beta-thalassemic patients it was detected only in one pool with a high read count, in SCD patients the HPgV-1 reads were identified in all but one pool. This finding shows the higher prevalence of HPgV-1 among SCD patients, which was also confirmed by HPgV-1 PCR detection in the individual patient samples. In SCD patients, the HPgV-1 prevalence (17.8%) was higher compared both to beta-thalassemia patients (6.7%) and blood donors (12.5%). HPgV-1 is transmitted mainly parenterally (14). However, the mean transfused volume was significantly lower in SCD patients compared to beta-thalassemia patients. This implies that parenteral transmission is not the only route for HPgV-1 infection. In support of this, several studies report alternative efficient HPgV-1 transmission routes such as sexual (15), horizontal, and vertical ones (16). In our study group, 37.5 % of HPgV-1-positive SCD patients were children, which could indicate a vertical/horizontal transmission. The remaining 62.7% of HPgV-1-positive SCD patients had a mean age of 34.8 years (range 19-57), indicating a possible combination of sexual and/or parenteral transmission. In contrast, in beta-thalassemia patients we can speculate that the lower HPgV-1 RNA prevalence is related to intrinsic pathophysiological protection mechanisms against viral infections.

It has been documented that the infection with HPgV-1 generates a humoral immune response mainly in the form of anti-E2 HPgV-1 IgG antibodies, which may protect against reinfections (17). It is possible that, due to the frequent blood transfusions, patients with beta-thalassemia have a higher number of HPgV-1 infections and for this reason, have been immunized against this virus. Nevertheless, this must be confirmed by serological studies comparing patients with SCD and beta-thalassemia major to investigate the seroprevalence to HPgV-1. We believe that in polytransfused patients, HPgV-1 transmission depends on an interplay of different factors including patient characteristics, serological status, age, sexual activity, lifestyle, and the number of transfusions received, which shape the different prevalence of HPgV-1 RNA in the patient groups studied.

Currently, the impact of commensal viruses on patients with multiple transfusions is unclear. Apart from some studies linking frequent TTV exposure to hepatic dysfunction or injury in polytransfused patients with beta-thalassemia (18), the vast majority of studies do not establish a cause-effect relationship (19,20). In our study, we observed that the most abundant TTV species were similar in the studied groups, although with different reads, being most abundant among patients with beta-thalassemia. On the contrary, HPgV-1 reads were more abundant in SCD patients. Consistent with this, we believe that the clinical impact of these viruses may be minimal on polytransfused patients but their presence could be used as a diagnostic marker for these diseases.

Our study had several limitations and the most important one is that we metagenomically analyzed pools of plasma samples and the individual distribution of the TTV types in individual patients was not known. Another limitation was that we did not quantify the viral load of all commensal viruses, which can also be related to the obtained metagenomic viral reads. Finally, we performed metagenomic identification of the virome in polytransfused patients, which only identifies viral genetic material and a causal relationship or mode of viral transmission cannot be determined. Therefore, further studies are needed to evaluate the impact of commensal viruses on blood transfusion and especially in patients who receive multiple transfusions as a part of their treatment.

In conclusion, multiple types of commensal viruses were detected in polytransfused patients with SCD and beta-thalassemia major, but in thalassemia patients the most abundant one was of anellovirus genotypes, whereas in SCD patients it was HPgV-1. This suggests that commensal viruses in human blood are not simple bystanders but might exert important functions and could be considered as specific disease and genetic markers. Nevertheless, more specific and complex studies are needed to investigate the abundance and functions of commensal viruses in patients receiving multiple transfusions and how they may influence hematologic treatment.
